# Hygienic and grooming behaviors in African and European honeybees—New damage categories in *Varroa destructor*

**DOI:** 10.1371/journal.pone.0179329

**Published:** 2017-06-16

**Authors:** Beatrice T. Nganso, Ayuka T. Fombong, Abdullahi A. Yusuf, Christian W. W. Pirk, Charles Stuhl, Baldwyn Torto

**Affiliations:** 1 International Centre of Insect Physiology and Ecology (*icipe*), Nairobi, Kenya; 2 Social Insects Research Group, Department of Zoology and Entomology, University of Pretoria, Pretoria, South Africa; 3 USDA/ARS—Centre for Medical, Agricultural and Veterinary Entomology, Gainesville, Florida, United States of America; University of Cologne, GERMANY

## Abstract

*Varroa destructor* is an ectoparasitic pest of honeybees, and a threat to the survival of the apiculture industry. Several studies have shown that unlike European honeybees, African honeybee populations appear to be minimally affected when attacked by this mite. However, little is known about the underlying drivers contributing to survival of African honeybee populations against the mite. We hypothesized that resistant behavioral defenses are responsible for the survival of African honeybees against the ectoparasite. We tested this hypothesis by comparing grooming and hygienic behaviors in the African savannah honeybee *Apis mellifera scutellata* in Kenya and *A*. *mellifera* hybrids of European origin in Florida, USA against the mite. Grooming behavior was assessed by determining adult mite infestation levels, daily mite fall per colony and percentage mite damage (as an indicator of adult grooming rate), while hygienic behavior was assessed by determining the brood removal rate after freeze killing a section of the brood. Our results identified two additional undescribed damaged mite categories along with the six previously known damage categories associated with the grooming behavior of both honeybee subspecies. Adult mite infestation level was approximately three-fold higher in *A*. *mellifera* hybrids of European origin than in *A*. *m*. *scutellata*, however, brood removal rate, adult grooming rate and daily natural mite fall were similar in both honeybee subspecies. Unlike *A*. *mellifera* hybrids of European origin, adult grooming rate and brood removal rate did not correlate with mite infestation levels on adult worker honeybee of *A*. *m*. *scutellata* though they were more aggressive towards the mites than their European counterparts. Our results provide valuable insights into the tolerance mechanisms that contribute to the survival of *A*. *m*. *scutellata* against the mite.

## Introduction

*Varroa destructor* Anderson and Trueman (Acari: Varoidae) is an ectoparasitic pest of the Western honeybee, *Apis mellifera* L. (Hymenoptera: Apidae). It feeds on the fat body of both immature and adult honeybees while transmitting lethal pathogens [1,2] causing severe physical and physiological injuries to individual honeybees [3]. In the absence of appropriate control measures, honeybee colonies heavily infested with the mites succumb within 1–2 years [2]. Interestingly, the mite is a relatively harmless pest on its native host, the Eastern honeybee *Apis cerana*, found mainly in Asia [4] which has efficient defensive mechanisms including hygienic and grooming behaviors to limit the mite’s reproduction in drone brood cells only which are generally less abundant than worker brood cells in a colony and do not occur throughout the year [5–7]. The mite is an invasive pest of the Western honeybee, *A*. *mellifera* which occurs elsewhere in the world (reviewed in [8]). Unlike *A*. *cerana*, the mite reproduces successfully in both worker and drone broods of *A*. *mellifera* [2]. Additionally, the absence of certain adaptive behavioral and physiological mechanisms that are present in its original host, has made the Western honeybee highly susceptible to the mite [9]. Pathogens associated with the mite are considered responsible for the decline of managed honeybee colonies especially in Europe and North America [10–13]. As a result, beekeepers in most of the affected countries substantially depend on in-hive chemical treatments to keep mite populations below economic thresholds so as to prolong survival of the colonies [2,10,14].

Previous studies carried out in Asia, Europe, South and North America have shown that *Apis cerana* and some populations of *Apis mellifera* have developed specific adaptive behaviors that enable them to co-exist with *Varroa* mite infestations [15]. These adaptive behaviors include hygienic and grooming behaviors, entombing of mites’ infested drone brood, restriction of mite’s reproduction in drone broods, suppression of the mite’s reproductive success, shorter post capping time and less attractive brood for mites.

In grooming behavior studies, an estimate of the percentage of damage inflicted on mites by honeybees is used as a measure of the bee’s grooming behavior [16]. This estimation can also be inferred from a damage classification scheme developed by Corrêa-Marques *et al*.,[17] comprising six different categories: a) damaged legs, b) hollow in the dorsal shield, c) carcass-empty dorsal shield, d) damage shield + damaged legs, e) hollow in the dorsal shield + damaged legs, and f) damaged shield. On the other hand, hygienic behavior is measured as the rate at which nurse bees remove dead or diseased brood [18].

Studies have shown that African honeybee populations survive mite infestation without requiring any managerial inputs by beekeepers. For example, field studies by various researchers demonstrated that survival of the South African Cape honeybee *A*. *m*. *capensis* against *Varroa* mite was linked to short post-capping stage, hygienic and grooming behaviors of this honeybee subspecies [19–21]. Likewise, survival of the savannah honeybee subspecies *A*. *m*. *scutellata* against the mite was found to be associated with reduced population growth, low viral prevalence, short post-capping stage, low fertility, fecundity and reproductive success of *Varroa* mite foundresses [19,22–24]. Interestingly, the East African population of *A*. *m*. *scutellata* has also been reported to survive *Varroa* mite parasitism, requiring no chemical treatment even when coexisting with other pathogens responsible for the losses of colonies in Europe and North America [25,26]. However, it is unknown whether survival of this specific African savannah honeybee population is associated with tolerance (the ability to limit the detrimental effects of the mite) or resistance (the ability to reduce the reproductive fitness of the mite) as part of its behavioral defense mechanisms or both [27]. To test the hypothesis that resistant defense mechanisms confer coping and survival strategies in this specific population of *Apis mellifera scutellata*, we compared the grooming and hygienic behaviors in this honeybee subspecies with those of *A*. *mellifera* hybrids of European origin found in the USA against the mite.

## Materials and methods

### Study sites

The study was conducted in Nairobi, Kenya from August—September 2015 (the cooler- dry season) and in Gainesville, Florida, United States of America in April 2016 (spring). These periods are characterized by reduced brood rearing in both savannah and European honeybees [28,29]. All the colonies were housed in standard Langstroth hives containing 3 to 4 brood combs and were not treated with acaricides to reduce mite infestations.

In Kenya, seventeen (17) queen right colonies were selected at two sites namely Kithimani (1°8' S, 37°25 E) (N = 10) and Kilimanbogo (1°8' S, 37°21' E) (N = 7) both located within the county of Machakos. These two apiaries were 7.4 Km apart and, contained colonies that originated from locally captured swarms. The colonies in this neighborhood host *A*. *m*. *scutellata* [26,29,30]. In Gainesville, Florida, USA, twenty colonies (20) were selected, with ten (10) each at the University of Florida apiary (29.62°38'N, 82.35°21'W) and USDA- ARS-CMAVE apiary (29.63°38'N, 82.36°21'W). These apiaries were ~ 1.6 Km apart and were bred from honeybee stocks purchased from local commercial queen breeders. The honeybee colonies in the USA were hybrids of different European subspecies (Ellis, personal communication).

### Molecular identification of *Varroa* mite strains

To confirm the strain of *Varroa* mites present in the savannah and hybrids of European honeybee colonies, two honeybee colonies were randomly selected among the colonies used at the individual apiaries in Kenya and the USA. Five living mites per colony (N = 5) were collected from adult worker honeybees on the brood area of the comb using the standard sugar-roll method [31]and preserved in 95% ethanol for DNA analysis at the USDA-ARS-CMAVE in Gainesville, Florida, USA. In total, eight mites were analyzed, that is, two mites per single colony in each apiary using the methods detailed below. Genomic DNA was extracted from individual mites using the DNeasy Tissue Kit (Qiagen, USA) per the manufacturer’s protocols for the spin-column protocol for Cultured Animal Cells with the following slight modifications: (i) all volumes were reduced to half; (ii) incubation was at 70°C for 1 hour; (iii) final elution was in 50 μL of Buffer AE. Nucleic acid concentrations were measured in each sample and three fragments from the cytochrome oxidase I (cox1), cytochrome oxidase III (cox3) and ATP synthase 6 (atp6) mitochondrial genes were amplified by polymerase chain reaction (PCR). The primers of these selected mitochondrial genes were purchased from Integrated DNA Technologies, Coralville, Iowa, USA [32]. The amplified gene fragment, primer name, primer sequences, product size base pairs (bp) and the annealing temperature for each fragment are presented in S1 Table. Reactions were carried out in 50 μl reactions containing 1X buffer, 0.05 U Taq polymerase (Invitrogen), 0.2 mM dNTPs, 0.4 μM of each oligonucleotide primer, 1.5 mM of MgCl2 and 1 μl of sample DNA. The positive control was gDNA from *Varroa destructor* samples identified at the study sites in Gainesville, Florida, USA. Cycling conditions involved initial denaturation at 94°C for 4 minutes, followed by 35 cycles of denaturation at 94°C for 30 seconds and annealing for 30 seconds and extension at 72°C for 1 minute. The amplicons were analyzed by gel electrophoreses on a 1.5% agarose gel run for 2 hours at 90 volts. PCR products were cleaned-up using the DNA Clean and Concentrator™-5 kit (Zymo Research) and bi-directionally sequenced by Macrogen (Maryland, USA). Sequences were edited with BioEdit Version 7.2.5.0 software [33]. Sequences obtained from individual mites were compared with those at National Center for Biotechnology Information (NCBI) using the online tool BLASTn to identify the *Varroa* mite strain. Species-level identification was determined when sequences exhibited ≥ 99% identity.

### Assessment of grooming behavior in honeybees of African and hybrids of European origin

Prior to the grooming behavior experiments, the level of infestations with *Varroa* mites on approximately hundred (100) adult worker honeybees in each colony was determined using the standard sugar-roll method [31]. The percentage of *Varroa* mite infestation rates in adult honeybees was determined by taking the number of *Varroa* mite collected divided by 100 adult worker honeybee and then multiplied by 100 [21,22].

Grooming behavior was assessed in the selected colonies in Kenya and USA using the screen bottom board method. Prior to the beginning of the study, the original bottom board of each colony was replaced with a modified bottom equipped with a retractable floor and covered with a screen mesh fine enough to permit only the passage of mites through its openings, thereby restricting the honeybees to further inflict damages on fallen mites. Cardboard white paper coated with sticky non-toxic petroleum jelly (Vaseline®) was smeared on the retractable floor to intercept falling mites and to protect them from being further damaged by predators such as ants, the small hive beetle and wax moth larvae. Natural fallen mites were collected every 24 hours from the debris on the bottom board using a fine Camel hair brush for a duration of 7 days and examined for injuries under a Leica S6E stereo microscope (×40 Magnification). The damaged mites were further grouped into different damage categories using the classification of mite’s damages [17]. The percentage of damaged mite in each colony was determined by dividing the number of damaged mites by the total number of dropped mites collected at the end of the collection period. The average daily natural fallen mite/per colony was determined by dividing the total number of natural fallen mites by the number of days mites were collected [22].

### Assessment of the source of physical damage on fallen mites in *A*. *m*. *scutellata* colonies

We investigated whether the recorded mite damages on the screen bottom boards of colonies were due to honeybee’s grooming behavior or other agents such as ants, small hive beetle or wax moth larvae. *Varroa* mites were collected from the savannah honeybee colonies using the standard sugar-roll method [31] from a subset of colonies at the Kithimani’s apiary in Kenya and freeze-killed at—80°C for 30 minutes. They were subsequently observed under a dissecting microscope to ensure that none was damaged before the beginning of the experiment. The dead, undamaged mites were marked on the dorsal shield with two permanent markers of different colors, blue and black. Three colonies (N = 3) were used for this experiment and grease oil was spread on the wooden platforms to restrict ants present from accessing the hives. In each colony, ten (10) black, marked mites were introduced on a white, glossy cardboard coated with sticky non-toxic petroleum jelly (Vaseline®) (to protect fallen mites from being further damaged by predators such as ants and wax moth larvae); and twenty (20) blue, marked mites were introduced in the brood area of one frame. Fallen mites were collected after 24 hours from the debris on the bottom board using a fine Camel hair brush and examined for injuries under a Leica S6E stereo microscope (×40 Magnification). The experiment was repeated three times.

### Assessment of hygienic behavior in honeybees of African and hybrids of European origin

Hygienic behavior was assessed in the selected colonies (N = 17) at each apiary in Kenya and in nine colonies (N = 9) at each apiary in the USA using the standard freeze-killed brood assay method with liquid nitrogen to freeze-kill young pupae (white- to purple-eyed stage with no cuticular tanning) as described by [34]. The number of fully removed freeze-killed brood cells from the test patch was recorded after a period of 24 and 48 hours and expressed as the percentage of the total brood containing cells at the start of the experiment.

### Ethical considerations

For field study in Kenya, written informed consents were obtained from the apiary owners. In the United States of America, we used apiaries managed by the USDA/ARS-Centre for Medical, Agricultural and Veterinary Entomology, Gainesville and the University of Florida.

### Statistical analyses

Statistical analyses were performed using R-Software version 3.2.5 [35]. In Kenya, a total of six colonies absconded during the experimental period including five from Kithimani and one from Kilimanbogo apiaries respectively. Consequently, these colonies could not be monitored for the entire duration of the experiment. In the USA, none of the colonies absconded during the entire monitoring period. Data from Kithimani and Kilimanbogo apiaries were pooled to obtain average total mite dropped, percentage of damaged mites, *Varroa* mite-infestation per 100 adult worker honeybees and the proportion of removed freeze-killed brood at 24 and 48 hours in the African savannah honeybee colonies. Likewise, data from the USDA-CMAVE and experimental farm of the University of Florida apiaries were pooled to obtain similar information in the colonies of honeybee hybrids of European origin. The count data were analyzed using generalized linear model (GLM) with log link and binomial distribution error to compare the factors: total number of fallen mites, *Varroa* mite-infestation level and the daily mites fall between both honeybee subspecies. Meanwhile, the proportion data were analyzed using generalized linear model (GLM) with logit link and binomial distribution error to compare the factors: percentages of damaged mites, different types of damages and freeze-killed brood removed at 24 and 48 hours between both honeybee subspecies. The effect of a factor for a GLM is reflected in the deviance (likelihood ratio test statistic) that has an appropriate chi-square distribution; hence the chi-square values are presented as test statistics. The Mann-Whitney-Wilcoxon test was used to compare the ratio of total natural fallen mite/*Varroa* mite-infestation level on adult worker honeybee between both honeybee subspecies. Spearman’s rank order correlation analysis was conducted to establish the existence of a relationship between the percentage of mite damage (overall and categorical damage types), total natural fallen mite, daily natural fallen mites/colony and brood removal (after 24 and 48 hours) to *Varroa* mite-infestation level on adult worker honeybee in each study site.

## Results

### Molecular identification of *Varroa* mite strains

*Varroa destructor* was the only *Varroa* mite species detected in the colonies of *A*. *m*. *scutellata* and *A*. *mellifera* hybrids of European origin and all the haplotypes belonged to the Korean strain (K1 haplotype).

### Assessment of grooming behavior in honeybees of African and hybrids of European origin

An infestation rate of 5 ± 1.4 mites/100 adult worker was recorded in the surviving African savannah honeybees which was significantly lower (~three-fold less) than the infestation rate in the susceptible hybrids of European origin honeybees at 14 ± 2.3 mites/ 100 adult worker (df = 32: F = 10.90; P = 0.001 < 0.05, Table 1).

**Table 1:** Mean ± standard error of mite infestation rates, daily mite fall and percentage of damaged mites on adult honeybee workers in colonies of *A*. *m*. *scutellata* and *A*. *mellifera* hybrids of European origin.

			Mean ± SE		
Sites	Honeybee species	Number of colonies	Mite infestation rate/100 adult worker bees (3 replicates/colony)	Daily mite fall/colony	% damaged mites
Kenya	*Apis mellifera scutellata*	14	5.0 ± 1.4	18 .1 ± 2.8	21.3 ± 1.7
USA	*Apis mellifera* hybrids of European origin	20	14 ± 2.3	15.8 ± 3.9	21.3 ± 2.4
**P-Value**^**a**^			0.001	0.60	0.84

a p values were calculated using the generalized linear model (GLM) with log or logit links

**Fig 1:**
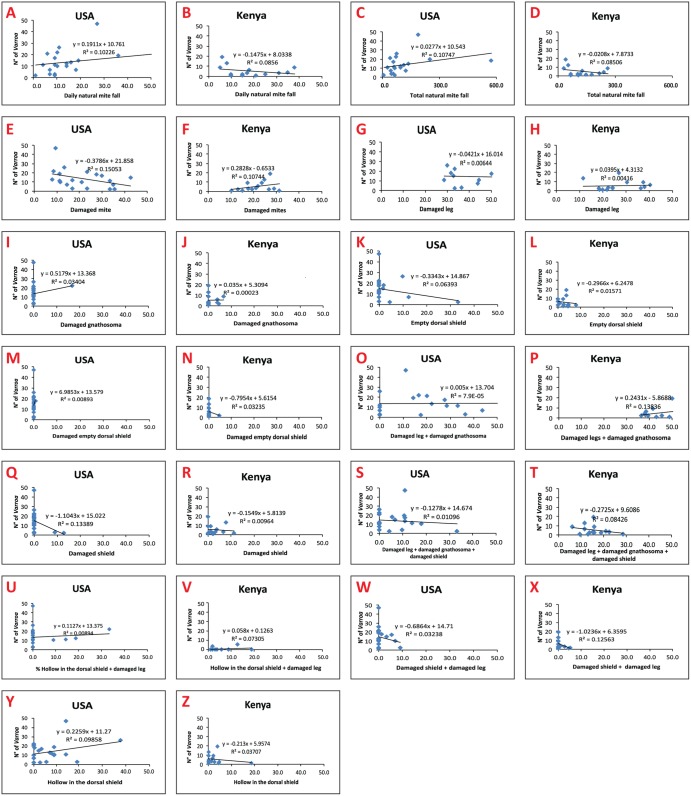
Correlation between daily natural mite fall, total natural mite fall, percentage damaged mites, different categories of damage to the mites and *Varroa*-mite infestation level per colony in honeybees of African and European origin in Kenya and USA respectively.

A total of 126.6 ± 3.2 natural fallen mites/colony was collected from the bottom boards of the African savannah honeybee (Kithimani; N = 8 colonies; Kilimanbogo; N = 6 colonies) compared to 110.6 ± 4.2 natural fallen mites/colony collected from the bottom boards of the hybrids European origin honeybees (USDA and University of Florida apiaries-Gainesville: N = 10 colonies in each), which were not significantly different (df = 236: F = 1.97; P = 0.16 > 0.05). Similarly, the daily natural mite fall/colony (df = 32: F = 0.28; P = 0.60 > 0.05) and the percentages of damaged mites (df = 236: F = 0.04; P = 0.84> 0.05) recorded in both honeybee subspecies colonies were not significantly different (Table 1). The ratio of total natural mite fall/mite infestation level was significantly higher in the African savannah honeybee colonies than those recorded in the hybrids of European origin honeybee colonies (W = 52, P = 0.002 < 0.05). There was no significant correlation between the daily natural mite fall/colony (Spearman’s rank correlation: r = -0.17, P = 0.55 > 0.05; Fig 1), total natural mite fall (Spearman’s rank correlation: r = -0.17, P = 0.57 > 0.05; Fig 1) and mite infestation level/colony in the African savannah honeybee colonies. In contrast, a significant and positive correlation was detected between the daily natural mite fall/colony (Spearman’s rank correlation: r = 0.48, P = 0.03 < 0.05; Fig 1), total natural mite fall (Spearman’s rank correlation: r = 0.47, P = 0.04 < 0.05; Fig 1) and mite infestation level/colony in the hybrids of European origin honeybee colonies. Also, there was no significant correlation between the percentage of damaged mites or the percentage of the different types of damages and the *Varroa*-infestation levels in the African savannah honeybees (Fig 1). A similar result was obtained in the hybrids of European origin honeybees with the exception that there was a significant negative correlation between damage to the mite’s dorsal shield or idiosoma and the adult worker honeybee mite infestation rates (Spearman’s rank correlation: r = -0.46, P = 0.04 < 0.05; Fig 1).

**Fig 2:**
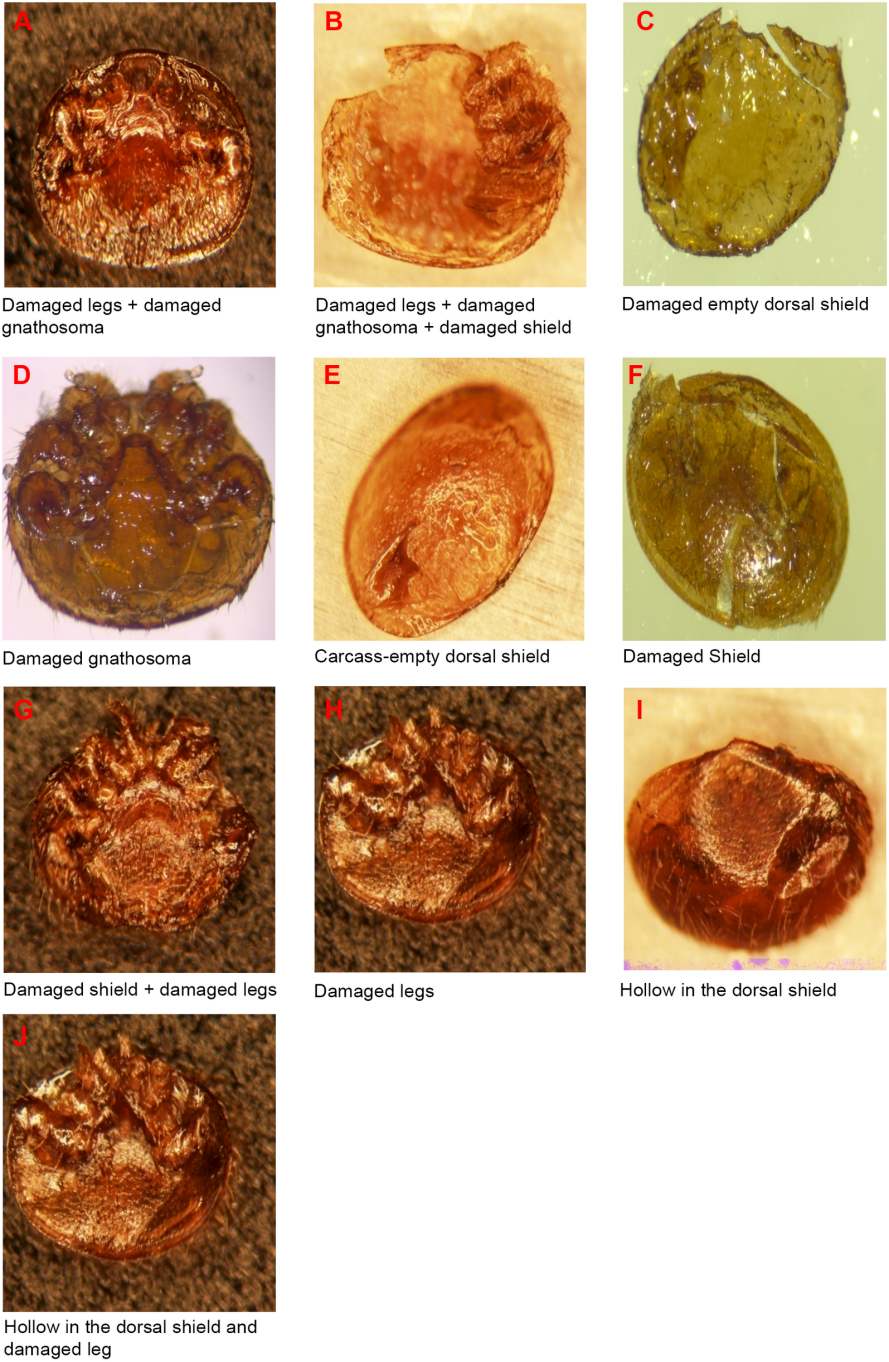
Photographs showing the different damage patterns in mature female *Varroa destructor* mite (×40 Magnification). (A and D) Damaged categories from literature [36–38]. (B and C) Additional damage categories reported in this study. (E-J) Previously known classification of damage to the mites reported by Corrêa-Marques *et al*., [17].

Different categories of damages to the mite were recorded in this study (Fig 2) including two additional previously undescribed damage categories to the mite namely; damaged empty dorsal shield and damaged legs + damaged gnathosoma + damaged shield (Fig 2B and 2C). These additional damage categories were present in colonies of both African savannah and hybrids of European origin honeybees (Table 2). Damaged leg (total or partial loss of one or more legs) was the predominant type of physical injury to the mite recorded in the hybrids of European origin honeybee colonies and this was significantly different from those found in the African savannah honeybee colonies (df = 236: F = 9.23; P = 0.003 < 0.05, Table 2). In the African savannah honeybee colonies, damaged legs + damaged gnathosoma was the predominant type of mite injury found and this was significantly different from those found in colonies of European hybrids (df = 236: F = 5.14; P = 0.02 < 0.05, Table 2).

**Table 2:** Percentages (mean ± SE) for the different categories of damages to *Varroa destructor* recorded in the colony debris of *A*. *m*. *scutellata* and *A*. *mellifera* hybrids of European origin in Kenya and USA respectively.

Category of damage	Kenya (%)	USA (%)	P- Value^a^
Damaged legs (DL) = total or partial loss of one or more legs	5.7 ± 0.6	10 ± 1.4	0.003
Hollow in the dorsal shield (HDS) = Depression in the dorsal shield	0.5 ± 0.2	2.3 ± 0.7	0.01
Empty dorsal shield (EDS)-carcass = mites that lacked all legs and all or almost all of the ventral shields, generally only the dorsal shield remained	0.6 ± 0.2	0.1 ± 0.7	0.01
Damaged shields (DS) = loss of dorsal shields, fissures in and loss pieces of the dorsal shield	0.6 ± 0.2	0.3 ± 0.2	0.001
Damaged shield + damaged legs (DS + DL)	0.2 ± 0.1	0.4 ± 0.2	0.46
Hollow in the dorsal shield + damaged legs (HDS + DL)	0.1 ± 0.1	0.4 ± 0.2	0.08
Damaged gnathosoma (DG) = loss of chelicerae and/or pedipalps	0.3 ± 0.1	0.1 ± 0.1	0.001
Damaged empty dorsal shield (DEDS) # = fissures in and loss of pieces of empty dorsal shield	0.1 ± 0.1	0.01 ± 0.01	0.44
Damaged legs + damaged gnathosoma (DL + DG) #	9.5 ± 0.7	6.1 ± 1.2	0.02
Damaged legs + damaged gnathosoma + damaged shield (DL + DG + DS) #	3.7 ± 0.4	1.6 ± 0.7	1.9e-10

#New damage categories observed in this study

a p values were calculated using the generalized linear model (GLM) with logit links

### Assessment of the source of physical damage on fallen mites in *A*. *m*. *scutellata* colonies

Out of the 90 marked, undamaged, dead mites introduced, four blue (6.7%) and four black (13.3%) marked mites were damaged, representing only 8.9% damaged mites of the overall mites introduced. The damages inflicted on the blue marked mites were likely caused by worker honeybees, while damages inflicted on the black marked mites may have been caused by other agents (e.g. wax moth larvae, small hive beetle adults and ants) found on the white, glossy cardboard fitted on the bottom board of colonies. We recorded only two types of damages to the mites in this experiment namely: damaged legs and damaged legs + damaged gnathosoma.

### Assessment of hygienic behavior in honeybees of African and hybrids of European origin

Brood removal rates at 24 and 48 hours were not significantly different between colonies of the African savannah (24 hours = 66.5 ± 8.3% and 48 hours = 81.0 ± 6.2%, mean ± SE) and the hybrids of European origin honeybees (24 hours = 59.1 ± 4.9% and 48 hours = 77.0 ± 3.9%, mean ± SE) (24 hours: F = 0.65, df = 27, P = 0.43 > 0.05; 48 hours: df = 27, F = 0.42, P = 0.52 > 0.05). There was a significant positive correlation between the mite infestation level of adult bees and the brood removal rate at 48 hours (Spearman’s rank correlation: r = 0.48, P = 0.04 < 0.05) though no correlation (Spearman’s rank correlation: r = 0.37, P = 0.13 > 0.05) was detected at 24 hours in the hybrids of European origin. In the African savannah honeybee colonies, there was no correlation between the mite infestation level of adult bees and the brood removal rate at 24 hours (Spearman’s rank correlation: r = -0.43, P = 0.19 > 0.05) and 48 hours (Spearman’s rank correlation: r = -0.30, P = 0.38 > 0.05).

## Discussion

### Grooming behavior

Our study suggests that European and African honeybees express similar grooming behavior since the percentage of damaged mites recorded on the bottom boards of both subspecies were similar. However, the phoretic mite numbers in both honeybee populations were different, approximately three-fold more in the European than in the African honeybee colonies. With more phoretic mites, one would expect to find more fallen and damaged mites on the bottom board; however, these values were not significantly different between both honeybee subspecies (Table 1). The absence of a significant correlation between the total natural mite fall, the percentage of damaged mites, the different categories of damage to the mite and *Varroa* mite-infestation levels suggests that grooming behavior may not explain the variability in *Varroa* mite-infestation levels recorded in the African and European honeybees. Moreover, these measures of grooming behavior (percentage of damaged mites or different categories of damage to mite) might not be sensitive enough to assess grooming behavior at the colony level as previously thought [17,37]. It is important to note that of the total mite population recorded on the bottom boards of honeybee colonies, not all mites which are groomed off by honeybees are damaged [38]. It is likely that damaged mites may also result from hygienic removal of infested capped brood by honeybees, and interactions with other arthropods in the colony such as the small hive beetle, wax moth and/or ants [16,38,39]. The ratio of total natural mite fall/mite infestation level, which represents a fraction of the total mite removed by honeybees off their bodies relative to the total mite population present in their colonies [40], was significantly higher in the African savannah honeybee colonies than those recorded in the colonies of their European counterparts. It appears that, the African savannah honeybee which maintains lower mite colony infestations displays a more efficient grooming behavior than its European counterpart. This finding corroborates results of previous studies which showed that colonies of *Varroa-*resistant *A*. *mellifera* subspecies also maintain lower mite loads and record a higher percentage of injured mites than their susceptible counterparts [41,42].

To further characterize the subspecies differences in grooming behavior, we analyzed the levels and patterns of damage in fallen mites using the previously known classification of damage to mites [17]. We found that the number of mites with only damaged legs was significantly higher in the European honeybee colonies than in colonies of the African counterpart. On the other hand, the numbers of mites with damaged legs and damaged gnathosoma were significantly higher in the African honeybee colonies than the European counterpart. This category of damage was first recorded in mites found in *A*. *m*. *carnica* colonies in Austria [36] but not included as a separate category of damage in the previous classification of damage to mites [17]. In the present study, the category described as legs and gnathosoma damage, was the second most frequent category of damage recorded in mites found in the European honeybee colonies. Moreover, we found two additional undescribed damage categories in mites namely damaged empty dorsal shield and damaged legs + damaged gnathosoma + damaged shield in both honeybee subspecies, and occurring more frequently in the African savannah honeybee than in the European honeybee. Overall, these results suggest that a higher aggressive behavior is displayed by the African savannah honeybee than by their European counterparts towards the mite. Taken together, these results provide additional insights into the grooming behavior of different subspecies of honeybees.

Based on recommendations for the control of *Varroa* mite in European honeybee colonies in the USA, interestingly, we observed that the *Varroa* infestation levels recorded in the savannah honeybee colonies were high enough to warrant miticide treatment [43]. Surprisingly, none of the colonies of *A*. *m*. *scutellata* used in the present study showed any signs of collapse. Typically, beekeepers in this region encountering such populations of *Varroa* mite in honeybee colonies neither administer any mite control measures [26] nor is done by beekeepers elsewhere on the rest of the African continent [44]. The *Varroa* infestation levels recorded in *A*. *m*. *scutellata* colonies in Kenya was similar to those recorded in colonies of the same honeybee subspecies found in South Africa [45] and no deleterious effects caused by the mites were reported [21,22,44,45]. The suppression of the mite reproductive output which translates into lower mite’s fertility, fecundity and lower reproductive rate (production of at least one viable, mated and mature female offspring) and the lower viral prevalence within honeybees and mites have been demonstrated to explain the slow rate of mite growth in *A*. *m*. *scutellata* colonies and their healthy appearance in colonies in South Africa [23,24]. Hence, other factors such as suppression of the mite’s reproductive success and/or lower viral prevalence within honeybees and mites might better explain the variability in the mite infestation levels observed between both *A*. *mellifera* subspecies [46,47]and should be evaluated in future studies.

### Hygienic behavior

Our study suggests a similar expression of the hygienic behavior trait in the European and African savannah honeybee since we recorded similar levels of brood removal rates in both honeybee populations. Our findings corroborate results of a previous study [47] which also found a similar expression of hygienic behavior between the Gotland mite-surviving and the local mite-susceptible honeybee populations in Sweden. Hygienic behavior appears not to explain the lower mite infestation rates observed in the savannah honeybee since we recorded no association between the brood removal rate and *Varroa* mite infestation levels. Our results differ from previously reported results [26] which found that colonies of *A*. *m*. *scutellata* which displayed higher levels of hygienic behavior had lower levels of *Varroa* mite infestation. These dissimilarities could be due to different climatic zones in which both studies were conducted and this might underline genotypic differences [48]. Nonetheless, hygienic behavior is known to be variable since it can be strongly influenced by environmental and in-hive factors [2]. Thus, the association between this behavior and *Varroa* mite loads in the savannah honeybee known to have a wide distribution range in Kenya [29] should be investigated in the future.

On the other hand, the significant positive correlation detected between mite infestation rate and brood removal at 48 hours in European honeybee colonies implies that more parasitized/ non-parasitized brood are removed under high *Varroa* parasitism. Our results suggest that hygienic behavior or brood removal rate is a response to the degree of diseased or parasitized brood found inside the brood cells. We expect that during spring period characterized by the early stages of brood production and lowest mite numbers in colonies [28], few mites will move inside the cells to reproduce, leading to a reduced removal of parasitized brood cells and vice-versa during mid or late summer period [28]. However, previous studies reported that European honeybee colonies bred for hygienic behavior were more efficient at removing *Varroa*-infested brood only under low mite parasitism and maintain lower mite loads on both adult honeybees and within worker brood cells than unselected colonies [18,49,50]. Under high parasitism (> 15% of both worker brood and adult honeybees), these colonies were found to fail to remove parasitized brood cells efficiently, requiring periodic miticide treatments to reduce their collapse [50]. As has been reported in breeding programs with Russian honeybees, hygienic and *Varroa*-sensitive hygienic honeybees in the USA, none of these honeybees have provided full protection for susceptible European honeybee colonies against *Varroa* mite infestation [3]. As such, they periodically require application of in-hive miticide to control the mite [3]. It appears that under high mite parasitism, honeybees invest significant resources into feeding their broods in order to obtain the next generation sub-optional worker honeybees than into other tasks such as grooming or hygienic behavior to remove infesting mites. Another explanation could be that, the build-up of large levels of odor cues released by parasitized broods which signal removal of diseased or parasitized brood cells in the colony might cause habituation and a reduction in receptor sensitivity to further detect odors [50–52]. Nevertheless, a long-term longitudinal study would help shed more light on the hygienic behavior in both the African savannah honeybee and their European counterparts.

## Conclusions

In host-parasite interactions, host tolerance is defined as the ability to limit the detrimental effects of the parasite while host resistance is the ability to reduce the reproductive fitness of the parasite [27]. In the present study, we found two additional undescribed damage categories in mites which occur more frequently in the African savannah honeybee than their European counterpart. Grooming behavior was better expressed in *A*. *m*. *scutellata* than in *A*. *mellifera* hybrids of European origin and hence, a potential tolerant mechanism displayed by the African savannah honeybee towards *V*. *destructor* attack. However, hygienic and grooming behaviors did not significantly differ between subspecies with respect to *Varroa* mite-infestation levels recorded suggesting that other resistant mechanisms such as suppression of mite reproductive success and/or lower viral prevalence within honeybees and mites might play an important role in honeybee responses to mite infestation.

## Supporting information

S1 Table Primers used for molecular analysis for identification of *Varroa* species and haplotype. Amplified gene fragment, product size base pairs (bp) and annealing temperatures (Ta) are indicated [32].
(DOCX)
