# Supplementary material for: Hygienic and grooming behaviors in African and European honeybees—New damage categories in Varroa destructor
Source: PLoS One. 2017 Jun 16;12(6):e0179329. doi: 10.1371/journal.pone.0179329 (PMC5473549; doi:10.1371/journal.pone.0179329)
Supplement: S1 Table — Amplified gene fragment, product size base pairs (bp) and annealing temperatures (Ta) are indicated [32]. (DOCX) [file pone.0179329.s001.docx]

| **Fragment** | **Primer name** | **Primer sequences (5’-3’)** | **Size (bp)** | **Ta (◦C)** |
| --- | --- | --- | --- | --- |
| *cox1* | 10KbCOIF1  6,5KbCOIR | CTT GTA ATC ATA AGG ATA TTG GAAC  AAT ACC AGT GGG AAC CGC | 929 | 52 |
| *atp6-cox3* | 6KbATP6F  16KbCOIIIR | GAC ATA TAT CAG TAA CAA TGAG  GAC TCC AAG TAA TAG TAA AACC | 818 | 52 |
